# Does a Raised Serum Troponin During a Severe Chronic Obstructive Pulmonary Disease Exacerbation Predict Future Cardiovascular Events?

**DOI:** 10.7759/cureus.79288

**Published:** 2025-02-19

**Authors:** Nyle Cockell, Nihal Billing, Praanesh Kumareshan, Thapas Nagarajan

**Affiliations:** 1 Undergraduate Medical Education, Salford Royal NHS Foundation Trust, Manchester, GBR; 2 School of Medicine, Faculty of Biology, Medicine and Health, University of Manchester Medical School, Manchester, GBR; 3 Respiratory Medicine, Manchester University NHS Foundation Trust, Manchester, GBR

**Keywords:** acute exacerbation of chronic obstructive pulmonary disease, cardiac biomarker, cardiac troponin, cardiovascular adverse events, cardiovascular event risk, copd: chronic obstructive pulmonary disease, high sensitivity troponin-i, major adverse cardiovascular event

## Abstract

Background

Cardiovascular (CV) complications are common in chronic obstructive pulmonary disease (COPD), particularly after acute exacerbations (AECOPD). Elevated cardiac biomarkers, such as high-sensitivity troponin I (hsTnI), indicate myocardial injury and commonly rise during AECOPD. While elevated serum troponin during severe AECOPD predicts mortality, the relationship between admission hsTnI levels and future CV event risk has not been investigated.

Aims and objectives

This study evaluated the prognostic value of admission serum troponin during severe AECOPD for future CV events, including new atrial fibrillation (AF), myocardial infarction (MI), or decompensated congestive cardiac failure (CCF) requiring intravenous diuretics.

Methods

This retrospective cohort study analyzed all patients admitted to a single center in 2022 with severe AECOPD and an admission hsTnI measurement. Patients were stratified by hsTnI levels (0-20ng/L and >20ng/L). The primary outcome was CV event incidence at 12 months, with secondary endpoints including event timing, type, and overall mortality.

Results

Patients with elevated hsTnI (n=37) had higher CV event incidence at 12 months compared to those with normal hsTnI (n=44) (24.3% vs 13.6%; OR 2.04, 95% CI 0.65-6.38). The hazard ratio (HR) for events was elevated but not statistically significant (HR 1.992, 95% CI 0.709-5.601, p=0.191). Raised hsTnI was associated with the greatest event risk at one month (OR 3.79 95% CI 0.38-38.1) and remained elevated over 12 months. Time to first event was also shorter in the elevated hsTnI group (3.0 vs 3.7 months, p=0.529).

CCF was the most frequent CV event (77% of all events), followed by MI and AF.​ Elevated hsTnI was associated with 12-month mortality (56.8% vs 36.3%; OR 1.83, 95% CI 0.75-4.48), although the HR did not reach statistical significance (HR 1.767, 95% CI 0.922-3.388, p=0.086).

Discussion

These findings indicate that elevated admission hsTnI during severe AECOPD is associated with increased CV event incidence, earlier time-to-event, and greater mortality over 12 months. Retrospective study design and opportunistic screening limited the ability to infer causality and statistical significance. Selection bias may have influenced the results from the clinical decision-making to measure hsTnI.​ Larger prospective studies with multivariate regression analysis are required to confirm these findings and address confounders.​

Conclusions

Our findings suggest that raised admission troponin levels are associated with CV events following severe AECOPD.  These patients may benefit from early CV risk assessment and preventative strategies.

## Introduction

Chronic obstructive pulmonary disease (COPD) refers to a group of long-term respiratory conditions, including chronic bronchitis and emphysema, that cause inflammation and narrowing of the airways. This results in airway obstruction and various breathing-related problems. A proportion of these patients will experience an acute exacerbation of COPD (AECOPD), a sustained episode of worsening symptoms such as shortness of breath, wheezing, sputum production, and sputum colour [1].

NICE recommends admitting patients with severe AECOPD in the presence of severe symptoms, such as severe dyspnea, acute confusion or impaired consciousness, cyanosis, oxygen saturation below 90%, and significant comorbidities such as insulin-dependent diabetes or cardiac disease [2]. Cardiovascular comorbidities complicate and increase mortality in patients with COPD, many of whom share risk factors such as smoking history and age. These factors are strongly correlated with the development of atherosclerosis and ischemic heart disease [3].

Patients experiencing moderate or severe exacerbations of COPD (AECOPD) are at increased risk for subsequent cardiovascular (CV) events [4,5]. The EXACOS-CV US study reported the greatest CV event incidence at one month (HR 1.34, 95% CI 1.23-1.46) and increased overall mortality (HR 1.79, 95% CI 1.58-2.04) following AECOPD [5]. While these risks decreased over time, they remained elevated for up to one year following the exacerbation. Subsequent exacerbations further increased these risks [5]. Graul et al. illustrated an early period of heightened risk in the initial 1-14 days (HR 3.19, 95% CI 2.71-3.76), particularly for arrhythmia and heart failure [3]. This window is considered critical for interventions aimed at preventing subsequent cardiovascular morbidity.

Several mechanisms likely contribute to myocardial injury during an acute exacerbation. This includes hypoxia, tachycardia, elevated pulmonary vascular resistance, and procoagulant state [6]. The etiology of these effects is thought to be multifactorial. During an exacerbation of COPD, airway inflammation can promote systemic effects including intravascular dysfunction and rupture of atherosclerotic plaques. Additionally, hypoxia and tachycardia caused by AECOPD can strain the heart, increasing blood pressure and facilitating thrombus formation [6,7].

Circulatory biomarkers of myocardial injury, such as high-sensitivity cardiac troponin T (hsTnT) and troponin I (hsTnI), are commonly elevated acutely in patients hospitalized with COPD exacerbations [8,9] and have been found to strongly predict in-hospital [10] and subsequent mortality [11,12]. It is important to note that a range of cardiac and non-cardiac conditions are associated with elevated troponin, including pulmonary embolism, renal failure, ischaemic stroke, and sepsis [13], which could independently contribute to short- and long-term mortality. Nevertheless, Washchki et al. demonstrated the predictive significance of a raised cardiac troponin even after adjusting for independence from known confounders and mortality predictors [12], which further indicates the prognostic ability of cardiac biomarkers in the AECOPD cohort.

While we understand elevated cardiac biomarkers to be a strong independent factor for subsequent mortality in COPD [14,15], their utility in predicting future cardiovascular events is less clear. No literature has yet reported a direct link between admission troponin levels and subsequent cardiovascular events, which makes this study unique. We evaluated the utility of admission serum troponin during severe AECOPD as a prognosticator for future CV events, which we defined as new atrial fibrillation (AF), myocardial infarction (MI) or admission with congestive cardiac failure (CCF) needing intravenous diuretics.

This article was previously presented as a poster presentation at the 2024 European Respiratory Society Congress in Vienna, Austria, on September 9, 2024.

## Materials and methods

Study design and setting

This single-center retrospective cohort study was conducted at a UK district general hospital. The inclusion period was from January to December 2022, following which demographic, clinical, and biochemical data were collected over 12 months. 

Population and sample size

Study Population

This study focused on adults, aged 18 years or older, who were admitted to the hospital with a severe AECOPD, for whom serum high-sensitivity troponin I (hsTnI) levels had been measured on admission. Retrospective sampling meant, the decision to sample serum hsTnI was at the discretion of the treating clinician.

Sample Size

Across the study period, a total of 1183 admissions with ICD-10 codes for COPD (J44.0, J44.1, J44.8, J44.9) were screened. From this, 81 patients met the inclusion criteria which formed the total sample size for the period. The study aimed to include all eligible patients across the specified timeframe, so no preliminary sample size calculation was performed. Convenience sampling was suitable for this retrospective study, as it relied on existing clinical data.

Inclusion and exclusion criteria

Inclusion Criteria

Patients aged 18 years and older with a consultant-confirmed diagnosis of severe acute exacerbation of COPD (AECOPD) requiring hospitalization were included in the study. Additionally, only those with a recorded serum high-sensitivity troponin I (hsTnI) level at the time of admission were considered eligible. A severe AECOPD was defined by the necessity of an emergency department visit or hospitalization and included patients with and without respiratory failure [16].

Exclusion Criteria

Patients were excluded if they did not have a diagnosis of AECOPD, did not require hospitalization due to a severe exacerbation, or lacked a recorded serum hsTnI level at admission.

These criteria were selected to identify the predictive value of serum troponin in a specified cohort of patients with severe AECOPD.

Data collection

Data were collected for all participants from hospital case notes and electronic health records. Relevant information included patient demographics (including age, gender, smoking history), severity and maintenance therapy of COPD, comorbidities, and clinical data from the admission. Comorbidities, spirometry results, and QRESEARCH risk estimator version 3 (QRISK3) scores were extracted from electronic primary care records. Where unavailable in the records, QRISK3 scores were calculated retrospectively at admission. Outcome data (mortality, CV event incidence) were collected from electronic hospital and primary care records.

Troponin Results

We recorded all participants’ admission serum hsTnI levels, where the reference range for our laboratory was between 0-20ng/L. Thus, the threshold elevated troponin value for the study was >20ng/L.

Outcomes

The primary endpoint was cardiovascular event incidence of any type (new atrial fibrillation (AF), myocardial infarction (MI), or admission with congestive cardiac failure (CCF) needing intravenous diuretics) at 12 months. Cerebrovascular events were excluded from outcome measures due to a lack of available data, on account of not being a stroke center. Secondary endpoints included overall mortality, co-existing CV morbidity, time to first event, and individual event type.

Data Security

The study was registered and approved by the Macclesfield District General Hospital Clinical Effectiveness Department for the Trust with reference number, RESP2302. The study was exempted from the ethical requirements due to its retrospective nature. Permission was gained from the Trust’s Information Governance Officer for publication of the material, providing no reference to the trust was made and data were unidentifiable.

Informed patient consent was waived on account of the retrospective study design. Patient-identifiable information was anonymized before analysis to maintain confidentiality. The data were password-protected on secure servers and accessible only to authorized personnel.

Data analysis

Statistical analyses were performed using IBM SPSS Statistics for Windows, Version 29.0, Armonk, NY: IBM Corp.

Descriptive Statistics

Parametric continuous variables such as age, forced expiratory volume in one second (FEV1), and QRISK3 score were presented as a mean and standard deviation. Non-parametric data, including systolic blood pressure and serum troponin, were synthesized into a median and interquartile range. Categorical values including sex, comorbidities, and clinical data were presented as frequencies and percentages.

Comparative Analysis

For continuous variables, normal distribution was assessed using the Shapiro-Wilk test. A t-test was used for parametric data, with homogeneity of variances evaluated using Levene’s test. When variances were estimated as equal (p>0.05), the t-test assuming equal variance was reported, otherwise Welch’s t-test was used. The Mann-Whitney U test was used for non-parametric data. Categorical variables were analyzed using Fisher’s exact test due to small sample sizes. Statistical significance was accepted for p-values less than 0.05.

Outcome Analysis

For all reported outcome data, 95% confidence intervals (CI) were estimated and a p-value of <0.05 was considered to indicate statistical significance. Univariate Cox regression was used to estimate hazard ratios (HR) for cardiovascular events and overall mortality outcomes. Kaplan-Meier survival curves were generated to visually represent the differences in outcome data across both groups. Odds ratios (OR) were calculated for cardiovascular event incidence at specific points in time.

To address the limitations of a small sample size, regression for covariates was not performed and results were interpreted for exploratory findings.

## Results

Demographic data

Between January and December 2022, a total of 81 participants hospitalized for severe AECOPD with recorded admission serum hsTnI levels were included in the study. As displayed in Table 1, 37 (46%) participants had elevated admission hsTnI levels (median 50ng/L, IQR 29-154ng/L), while 44 (54%) participants had troponin within the laboratory reference range (0-20ng/L). The mean age of the study cohort was 77.8 years (SD 11.9 years). Although the mean age in the normal troponin group was slightly lower (76.7 years, SD 11.3 years) compared to the raised troponin group (77.8 years, SD 11.9 years), the difference was not statistically significant (p=0.677). Gender distribution was broadly matched across both groups with 17 (39%) males in the normal troponin group, and 17 (46%) males in the elevated troponin group (p=0.652).

**Table 1:** Demographics of the study participants. Independent samples t-test was used for parametric data, and Mann-Whitney U test for non-parametric data or where parametric assumptions were not met. Fisher’s exact test was utilized for categorical data. Two-sided p-values are reported. hsTnI: high-sensitivity troponin I; CI: confidence interval; SD: standard deviation, IQR: interquartile range.

Factor	hsTnI (ng/L) 0.0–20.0 (n=44)	hsTnI (ng/L) >20.0 (n=37)	Test statistic	p-value
Demographics				
Male, n (%)	17 (39)	17 (46)	N/A	0.652
Age (years), mean (SD)	76.7 (11.3)	77.8 (11.9)	t=-0.419	0.677
Smoking history and COPD severity				
Current smoker, n (%)	14 (32)	13 (35)	N/A	0.815
Former smoker, n (%)	28 (64)	22 (59)	N/A	0.819
Never smoker, n (%)	2 (5)	2 (5)	N/A	1.000
Long-term oxygen, n (%)	2 (5)	3 (8)	N/A	0.656
Pack years, median (IQR)`	33 (20-60)	32.5 (25-50)	U = 517	0.969
FEV1 % of predicted, mean (SD)	46.8 (16.5)	55.0 (20.6)	t = -1.617	0.112
Previous medical history				
Coronary artery disease, n (%)	12 (27)	11 (30)	N/A	0.810
Prev CABG or PCI, n (%)	1 (2)	0	N/A	1.000
Atrial fibrillation, n (%)	4 (9)	10 (27)	N/A	0.042
Complete heart block, n (%)	0	1 (3)	N/A	0.457
Left bundle branch block, n (%)	0	2 (5)	N/A	0.206
Heart failure, n (%)	7 (16)	7 (19)	N/A	0.774
Pulmonary hypertension, n (%)	0	2 (5)	N/A	0.206
Pulmonary embolus, n (%)	2 (5)	4 (11)	N/A	0.404
Valvular disease, n (%)	2 (5)	1 (3)	N/A	1.000
Peripheral vascular disease, n (%)	0	3 (8)	N/A	0.091
Hypertension, n (%)	27 (61)	20 (54)	N/A	0.652
Diabetes mellitus, n (%)	9 (20)	10 (27)	N/A	0.601
Cerebrovascular event, n (%)	3 (7)	2 (5)	N/A	1.000
Chronic kidney disease stage 3+, n (%)	9 (20)	11 (30)	N/A	0.439
Rheumatoid arthritis, n (%)	2 (5)	2 (5)	N/A	1.000
Migraine, n (%)	1 (2)	2 (5)	N/A	0.590
Severe mental Illness, n (%)	13 (30)	10 (27)	N/A	1.000
Atypical antipsychotics, n (%)	3 (7)	0	N/A	0.246
Long-term steroids, n (%)	0	1 (3)	N/A	0.457
Serum cholesterol, median (IQR)	2.95 (2.54-3.60)	3.80 (2.60-4.20)	U = 157	0.058
Systolic blood pressure, median (IQR)	133.5 (115-151)	130 (120-143.5)	U = 700	0.720
QRISK3 score, mean (SD)	29.3 (11.9)	35.6 (13.8)	t = -2.215	0.030
Medication history pre-admission				
Beta blocker, n (%)	17 (39)	13 (35)	N/A	0.819
Aspirin, n (%)	15 (34)	7 (19)	N/A	0.142
Clopidogrel, n (%)	5 (11)	3 (8)	N/A	0.721
Ticagrelor, n (%)	0	0	N/A	-
Warfarin, n (%)	1 (2)	0	N/A	1.000
DOAC, n (%)	1 (2)	9 (24)	N/A	0.004
Statin, n (%)	21 (48)	20 (54)	N/A	0.657
ACEi/ARB, n (%)	14 (32)	15 (41)	N/A	0.488
Medication history post-admission				
Beta Blocker, n (%)	16 (36)	15 (41)	N/A	1.000
Aspirin, n (%)	15 (34)	9 (24)	N/A	0.630
Clopidogrel, n (%)	5 (11)	4 (11)	N/A	1.000
Ticagrelor, n (%)	0	0	N/A	-
Warfarin, n (%)	1 (2)	0	N/A	1.000
DOAC, n (%)	2 (5)	13 (35)	N/A	<0.001
Statin, n (%)	20 (45)	19 (51)	N/A	0.659
ACEi/ARB, n (%)	14 (32)	17 (46)	N/A	0.252
Admission clinical data				
Rescue pack prior, n (%)	8 (18)	4 (11)	N/A	0.532
New atrial fibrillation, n (%)	1 (2)	3 (8)	N/A	0.327
Decompensated heart failure, n (%)	4 (9)	5 (14)	N/A	0.725
Myocardial infarction, n (%)	0	4 (11)	N/A	0.040
Oxygen therapy, n (%)	18 (41)	24 (65)	N/A	0.045
Non-invasive ventilation, n (%)	10 (22)	9 (24)	N/A	1.000
Intensive care admission, n (%)	2 (5)	2 (5)	N/A	1.000
Inhaler therapy changed, n (%)	2 (5)	3 (8)	N/A	0.656
Laboratory data				
Eosinophils 10^9/L, median (IQR)	0.085 (0.04-0.19)	0.04 (0.02-0.10)	U=595	0.037
Troponin ng/L, median (IQR)	9 (5.5-14)	50 (29-154)	U=0	<0.001

Smoking History and COPD Severity

No statistically significant differences in smoking history were observed between groups (current smoker: p 0.815; ex-smoker: p=0.819; never smoked: p=1.000). Smoking pack years were comparable, with a median of 33 years (IQR 20-60 years) in the in-range hsTnI arm and 32.5 years (IQR 25-50 years) in the raised hsTnI arm (p=0.969). Spirometry data revealed a mean FEV1% predicted of 46.8% (SD 16.5) in the in-range hsTnI arm versus 55.0% (SD 20.6) in the raised hsTnI group, but this was not statistically significant (p=0.112). Long-term oxygen therapy use was also similar between groups (5% vs 8%, p=0.656).

Past Medical History

There were no statistically significant differences in major comorbidities between the two groups, including coronary artery disease (27% vs 30%, p=0.810), heart failure (16% vs 19%, p=0.774), or diabetes mellitus (20% vs 27%, p=0.601). However, atrial fibrillation was more prevalent in the raised hsTnI arm (27%), compared to the in-range hsTnI group (9%), with a statistically significant difference (p=0.042). Furthermore, the raised hsTnI group had a significantly higher mean QRISK3 score (29.3, SD 11.9), than the in-range hsTnI group (29.3, SD 11.9; p=0.030).

Admission Clinical Data

Significant clinical findings during index admission included myocardial infarction, observed in four patients (11%) in the raised hsTnI arm but in none of the in-range hsTnI arm (p=0.040) in Table 1. Between groups, there were no significant differences in new atrial fibrillation (2% vs 8%, p=0.327) or decompensated heart failure incidence (9% vs 14%, p=0.725). These admission-related events were excluded from outcome analysis, in order to examine for associations with future CV events.

Outcome data

Cardiovascular Event Incidence

**Figure 1:**
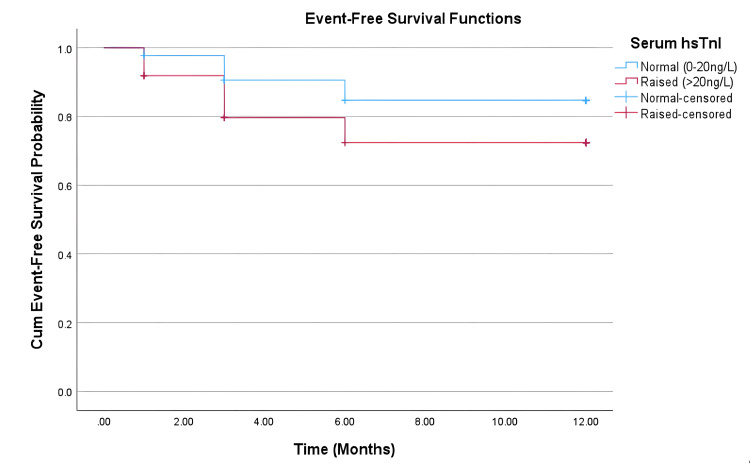
Kaplan-Meier curve for cardiovascular event-free survival probability. Patients were censored at the time of death or at the end of the 12-month period. hsTnI: high-sensitivity troponin I.

Hazard ratios calculated through univariate Cox regression analysis indicated increased cardiovascular risk in the elevated hsTnI arm, compared to the in-range hsTnI arm (HR 1.992, 95% CI 0.709-5.601) but did not reach statistical significance (p=0.191) (Table 2). This trend is visually represented in Figure 1.

**Table 2:** Univariate Cox regression analysis of cardiovascular event incidence across all timeframes. hsTnI: high-sensitivity troponin I; CI: confidence interval.

Variable	Hazard Ratio (HR)	95% CI	p-value
Elevated hsTnI	1.992	0.709-5.601	0.191

**Figure 2:**
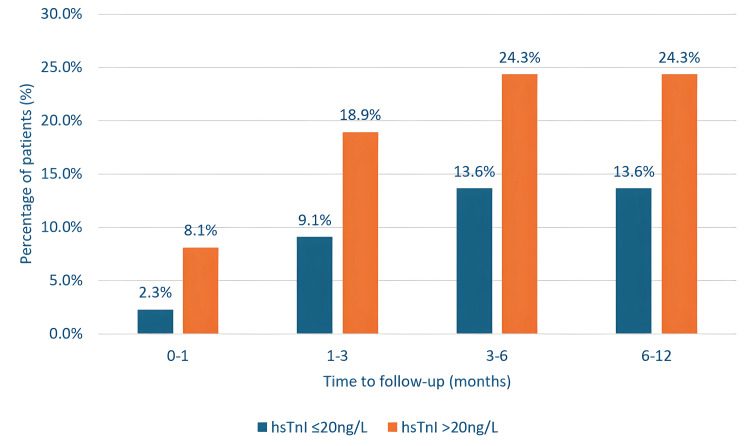
Comparison of first cardiovascular event diagnosis across all participants in the study at 12 months. hsTnI: high-sensitivity troponin I.

Figure 2 shows that at both 6 and 12-month follow-ups, 24% with elevated hsTnI had experienced a CV event, compared to 14% in the normal hsTnI group (OR 2.04 95% CI 0.65-6.38).

Table 3 demonstrates the odds ratios for CV events in the raised hsTnI arm, which were greatest within one month, remained elevated over the 12-month follow-up period, and decreased over time.

**Table 3:** Cardiovascular event incidence over time. hsTnI: high-sensitivity troponin I; CI: confidence interval.

CV event incidence	hsTnI (ng/L) >20.0 (n=37)	hsTnI (ng/L) 0.0–20.0 (n=44)	Odds ratio (95% CI)
1-month, n (%)	3 (8.1)	1 (2.3)	3.79 (0.38-38.1)
3-month, n (%)	7 (18.9)	4 (9.1)	2.33 (0.63-8.70)
6-month, n (%)	9 (24.3)	6 (13.6)	2.04 (0.65-6.38)
12-month, n (%)	9 (24.3)	6 (13.6)	2.04 (0.65-6.38)

Time to First Cardiovascular Event

Table 4 indicates that the mean time to the first CV event was shorter in the raised troponin group (3.0 months, SD 1.9) compared to the normal troponin group (3.7 months, SD 2.0), but did not reach statistical significance (p=0.529). Across the entire cohort, most first CV events (73%) occurred within three months of index AECOPD (mean 3.3 months, SD 1.9).

**Table 4:** Mean time to first cardiovascular event. Mann-Whitney U test with two-sided p-value is reported. hsTnI: high-sensitivity troponin I; CI: confidence interval; SD: standard deviation.

	hsTnI (ng/L) >20.0 (n=37)	hsTnI (ng/L) 0.0–20.0 (n=44)	Test statistic	p-value
Mean time to first event, months (SD)	3.0 (1.9)	3.7 (2.0)	U=21.5	0.529

Type of First Cardiovascular Event

**Figure 3:**
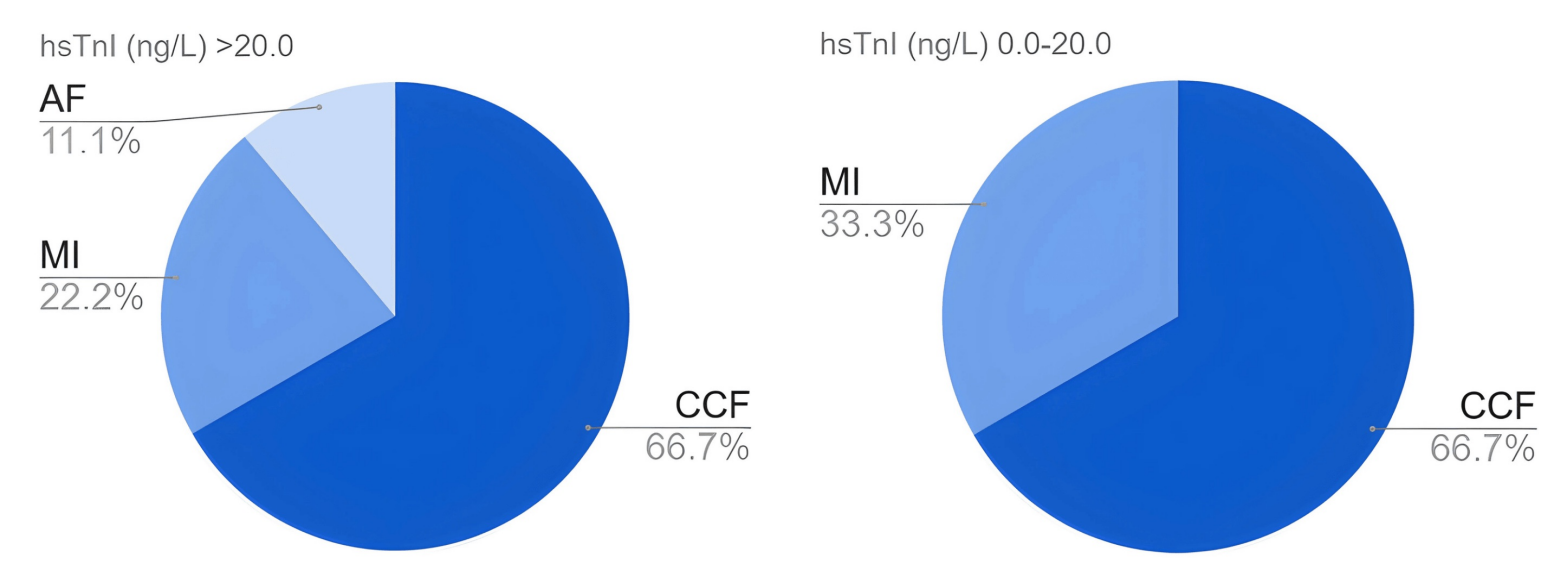
Proportion of first cardiovascular event diagnoses at 12 months. hsTnI: high-sensitivity troponin I; CCF: congestive cardiac failure; MI: myocardial infarction; AF: atrial fibrillation.

From the aggregated 15 participants who experienced a cardiovascular event, the majority (66.7%) had congestive cardiac failure as their first event, as shown in Figure 3.

Table 5 highlights two myocardial infarctions captured as the first event in each group and a single new atrial fibrillation diagnosis in the elevated hsTnI arm at 12 months. The raised hsTnI arm had a more frequent occurrence of congestive cardiac failure (CCF) admissions (16.2%) than the normal troponin arm (9.1%) (OR 1.94, 95% CI 0.50-7.46).

**Table 5:** Comparison of first cardiovascular event diagnosis across all participants in the study at 12 months. CV: cardiovascular; hsTnI: high-sensitivity troponin I; CI: confidence interval; CCF: congestive cardiac failure; MI: myocardial infarction; AF: atrial fibrillation.

First CV event encountered	hsTnI (ng/L) >20.0 (n=37)	hsTnI (ng/L) 0.0–20.0 (n=44)	Odds ratio (95%CI)
CCF, n (%)	6 (16.2)	4 (9.1)	1.94 (0.50-7.46)
MI, n (%)	2 (5.4)	2 (4.5)	1.2 (0.16-8.96)
AF, n (%)	1 (2.7)	0	3.66 (0.14-92.5)

Type of All Cardiovascular Events

**Figure 4:**
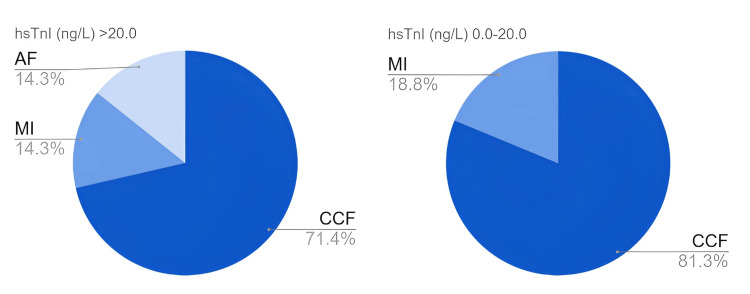
All cardiovascular events at 12 months. * *hsTnI: high-sensitivity troponin I; CCF: congestive cardiac failure; MI: myocardial infarction; AF: atrial fibrillation.

Figure 4 and Table 6 summarise all 30 CV events recorded across the cohort at 12 months with CCF decompensations comprising 77%. Statistical analysis was not performed for CCF decompensations due to multiple admissions per participant. Myocardial infarction occurred in three participants (7%) with normal hsTnI and in two patients (5%) with raised hsTnI (OR 0.78, 95% CI 0.12-4.94). Notably, new AF diagnosis occurred exclusively in the elevated hsTnI group (5.4%, OR 6.27, 95% CI 0.29-134.8).

**Table 6:** All cardiovascular events at 12 months. CV: cardiovascular; hsTnI: high-sensitivity troponin I; CI: confidence interval; CCF: congestive cardiac failure; MI: myocardial infarction; AF: atrial fibrillation.

Total number of CV events encountered	hsTnI (ng/L) >20.0 (n=37)	hsTnI (ng/L) 0.0–20.0 (n=44)	Odds ratio (95% CI)
CCF, n	10	13	-
MI, n (%)	2 (5.4)	3 (6.8)	0.78 (0.12-4.94)
AF, n (%)	2 (5.4)	0	6.27 (0.29-134.8)

Mortality

**Figure 5:**
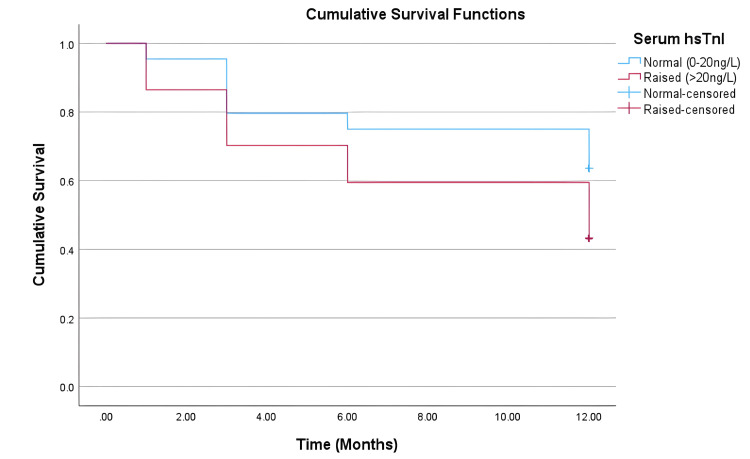
Kaplan-Meier curve for overall survival probability. Patients were censored at the end of the 12-month period. hsTnI: high-sensitivity troponin.

Elevated admission hsTnI favored any-cause mortality across the study period (HR 1.767, 95% CI 0.922-3.388, p=0.086), as summarised in Table 7. This trend is reflected in the Kaplan-Meier curve (Figure 5).

**Table 7:** Univariate Cox regression analysis of any-cause mortality across all timeframes. hsTnI: high-sensitivity troponin I; CI: confidence interval.

Variable	Hazard ratio (HR)	95% CI	p-value
Elevated hsTnI	1.767	0.922-3.388	0.086

Table 8 further highlights higher mortality in patients with raised hsTnI across all timeframes, with 56.7% mortality at 12 months versus 36.4% in the normal hsTnI arm (OR 1.83 95% CI 0.75-4.48) (Figure 2). The greatest risk was observed at one month (OR 3.28, 95% CI 0.60-18.0).

**Table 8:** Mortality of the participants. hsTnI: high-sensitivity troponin I; CI: confidence interval.

Mortality at follow-up	hsTnI (ng/L) >20.0 (n=37)	hsTnI (ng/L) 0.0–20.0 (n=44)	Odds ratio (95% CI)
1-month, n (%)	5 (13.5%)	2 (4.6%)	3.28 (0.60-18.0)
3-month, n (%)	11 (29.7%)	9 (20.5%)	1.65 (0.60-4.55)
6-month, n (%)	15 (40.5%)	11 (25.0%)	2.04 (0.79-5.27)
12-month, n (%)	21 (56.8%)	16 (36.3%)	2.30 (0.94-5.62)

## Discussion

Our findings indicate that raised admission hsTnI levels during a severe AECOPD are associated with an increased risk of future cardiovascular events across all timeframes up to 12 months, with a hazard ratio of 1.992 (95% CI 0.709-5.601, p=0.191). While wide confidence intervals reflect the uncertainty inherent within the study design, these hypothesis-generating findings have advanced our understanding of potential predictive factors for cardiovascular risk in the COPD population.

The greatest odds for a CV event were observed within the first month (OR 3.79, 95% CI 0.38-38.1) but remained elevated at 12 months. This aligns with existing literature which describes a heightened risk of cardiovascular complications immediately after an AECOPD, meaning this early post-exacerbation period may be a critical window for intervention [4,5]. Importantly, our data demonstrated a shorter time to the first event (3.0 months, SD 1.9) than the normal hsTnI group (3.7 months, SD 2.0), but did not reach statistical significance (p=0.529). Again, this suggests the need for heightened surveillance and preventative strategies in this period and further characterization of patients at greatest risk.

These results are consistent with previous studies which identified decompensated heart failure as the most frequently reported event following a COPD exacerbation, followed by myocardial infarction [4,5]. This finding underscores the interplay between COPD exacerbations and acute cardiac complications, supporting the growing body of evidence advocating for the integration of cardiovascular prevention into COPD care.

Our study has shown elevated mortality in the raised troponin group (HR 1.767, 95% CI 0.922-3.388, p=0.086), but the lack of statistical significance warrants further investigation in larger, adequately powered studies to validate these findings. Despite this, this study has further illustrated an association and potential utility for an elevated serum troponin to predict adverse survival outcomes in this population.

In summary, these findings further support the integration of cardiovascular risk assessment into routine care for COPD patients, particularly in the early post-exacerbation phase. Current guidelines primarily address general cardiovascular risk prevention and appear to overlook the complexities of COPD comorbidity. Given the apparent risk of future CV events, particularly decompensated heart failure, targeted interventions addressing modifiable risk factors and commencing secondary prevention could mitigate CV morbidity in these patients.

Limitations

The retrospective nature of the study inherently limits the ability to establish causality between elevated cardiac biomarkers and subsequent cardiovascular events. Additionally, the opportunistic sampling of serum troponin resulted in a relatively small cohort size which limited statistical power and the ability to identify significant differences across many outcomes. Despite these challenges, this study satisfied the aim of identifying a potential association between hsTnI and cardiovascular outcomes, providing a foundation for future prospective studies. These should be powered to confirm causality.

The decision to measure serum troponin was guided by clinical judgment, likely leading to the over-representation of patients with more severe symptoms or clinical instability. This selection bias likely contributed to the high observed mortality across both study arms. Our observed 12-month mortality (56.7% in the raised hsTnI arm; 36.3% in the normal hsTnI arm) exceeds the previously reported rates of 26-43% for severe AECOPD hospitalizations [17,18]. The single-center design also limits the generalizability of these findings in wider healthcare systems with variations in COPD guidelines, as well as diversity in socioeconomic determinants of cardiovascular risk between populations [19]. A prospective multi-center study design is needed to characterize the wider AECOPD patient cohort and allow causal inference for conclusions.

Excluding myocardial infarctions diagnosed during admission from outcome data aimed to minimize selection bias in the raised hsTnI group, reflecting obscurity in whether the AECOPD triggered the CV event, or vice versa. While this decision avoided confounding, it may have underestimated the true event rate during the critical first two weeks of greatest event risk [3]. Furthermore, the clinical sequelae from a myocardial infarction include further cardiovascular events and death [20], which may have confounded the predictive validity for future CV events and mortality in the raised troponin cohort.

The raised hsTnI arm had significantly more participants with known atrial fibrillation, itself a recognized cause of raised serum troponin associated with future cardiovascular events, including stroke, heart failure, and mortality [21]. This imbalance likely explains inter-arm differences in pre-admission oral anticoagulant use and QRISK3 scores. However, low CV event numbers from the opportunistic screening design limited the events-per-variable and prevented multivariate regression analysis, which could have better adjusted for these confounders.

This study was further limited by cerebrovascular event records being sometimes unavailable for analysis, as patients with suspected stroke were redirected to local stroke centers instead. Difficulties encountered with capturing these outcomes led to their exclusion from the study. While ischemic strokes have been reported to be the least frequently captured major CV event in the exacerbating COPD cohort [4,5], their exclusion here may have underestimated the total cardiovascular burden in this cohort. Furthermore, asymptomatic or misdiagnosed CV events may have led to underreporting or overreporting of outcome measures.

## Conclusions

Despite these limitations, the study has successfully elucidated a relationship between elevated admission troponin levels and subsequent cardiovascular event risk and mortality. We have also further highlighted how the first few months following an AECOPD represent a period of heightened cardiovascular risk. Due to limitations, these findings alone are insufficient to recommend routine serum troponin screening for all patients admitted with AECOPD. Nevertheless, these trends suggest a clinically relevant association, to provide justification for prioritizing large-scale prospective investigation into the utility of hsTnI as a marker for cardiovascular risk stratification in this population. By developing comprehensive risk stratification models that include hsTnI and other prognosticators, future research could aim to establish primary or secondary preventative interventions tailored to COPD patients with elevated cardiovascular risk.

## References

[1] Crisafulli E, Barbeta E, Ielpo A, Torres A (2018). Management of severe acute exacerbations of COPD: an updated narrative review. Multidiscip Respir Med.

[2] National Institute for Health and Care Excellence (NICE) (2025). National institute for health and care excellence (NICE). Chronic obstructive pulmonary disease. CKS. Chronic obstructive pulmonary disease. CKS.

[3] Graul EL, Nordon C, Rhodes K (2024). Temporal risk of nonfatal cardiovascular events after chronic obstructive pulmonary disease exacerbation: a population-based study. Am J Respir Crit Care Med.

[4] Calabria S, Ronconi G, Dondi L (2024). Cardiovascular events after exacerbations of chronic obstructive pulmonary disease: Results from the EXAcerbations of COPD and their OutcomeS in CardioVascular diseases study in Italy. Eur J Intern Med.

[5] Daniels K, Lanes S, Tave A (2024). Risk of death and cardiovascular events following an exacerbation of COPD: the EXACOS-CV US study. Int J Chron Obstruct Pulmon Dis.

[6] Li XF, Wan CQ, Mao YM (2022). Analysis of pathogenesis and drug treatment of chronic obstructive pulmonary disease complicated with cardiovascular disease. Front Med (Lausanne).

[7] Yang HM, Ryu MH, Carey VJ (2024). Chronic obstructive pulmonary disease exacerbations increase the risk of subsequent cardiovascular events: a longitudinal analysis of the COPDGene study. J Am Heart Assoc.

[8] Patel AR, Kowlessar BS, Donaldson GC (2013). Cardiovascular risk, myocardial injury, and exacerbations of chronic obstructive pulmonary disease. Am J Respir Crit Care Med.

[9] Søyseth V, Kononova N, Neukamm A, Holmedahl NH, Hagve TA, Omland T, Einvik G (2021). Systemic inflammation induced by exacerbation of COPD or pneumonia in patients with COPD induces cardiac troponin elevation. BMJ Open Respir Res.

[10] Baillard C, Boussarsar M, Fosse JP (2003). Cardiac troponin I in patients with severe exacerbation of chronic obstructive pulmonary disease. Intensive Care Med.

[11] Høiseth AD, Neukamm A, Karlsson BD, Omland T, Brekke PH, Søyseth V (2011). Elevated high-sensitivity cardiac troponin T is associated with increased mortality after acute exacerbation of chronic obstructive pulmonary disease. Thorax.

[12] Waschki B, Alter P, Zeller T (2020). High-sensitivity troponin I and all-cause mortality in patients with stable COPD: an analysis of the COSYCONET study. Eur Respir J.

[13] Korff S, Katus HA, Giannitsis E (2006). Differential diagnosis of elevated troponins. Heart.

[14] Vergaro G, Aimo A, Januzzi JL Jr (2022). Cardiac biomarkers retain prognostic significance in patients with heart failure and chronic obstructive pulmonary disease. J Cardiovasc Med (Hagerstown).

[15] Nilsson U, Mills NL, McAllister DA (2020). Cardiac biomarkers of prognostic importance in chronic obstructive pulmonary disease. Respir Res.

[16] (2025). Global initiative for chronic obstructive lung disease (GOLD). 2023 GOLD report. https://goldcopd.org/2023-gold-report-2/.

[17] García-Sanz MT, Cánive-Gómez JC, Senín-Rial L, Aboal-Viñas J, Barreiro-García A, López-Val E, González-Barcala FJ (2017). One-year and long-term mortality in patients hospitalized for chronic obstructive pulmonary disease. J Thorac Dis.

[18] Donaldson GC, Wedzicha JA (2006). COPD exacerbations .1: Epidemiology. Thorax.

[19] Borkowski P, Borkowska N, Mangeshkar S, Adal BH, Singh N (2024). Racial and socioeconomic determinants of cardiovascular health: a comprehensive review. Cureus.

[20] Wang Y, Li J, Zheng X (2018). Risk factors associated with major cardiovascular events 1 year after acute myocardial infarction. JAMA Netw Open.

[21] Parwani AS, Boldt LH, Huemer M (2013). Atrial fibrillation-induced cardiac troponin I release. Int J Cardiol.

